# Особенности клинического течения и частоты осложнений в кластерах сахарного диабета 2 типа у пациентов, не получающих инсулинотерапию

**DOI:** 10.14341/probl13259

**Published:** 2023-11-11

**Authors:** И. А. Бондарь, О. Ю. Шабельникова

**Affiliations:** Новосибирский государственный медицинский университет; Новосибирский государственный медицинский университет

**Keywords:** сахарный диабет 2 типа, кластеры, осложнения диабета, инсулинорезистентность, HOMA-IR, HOMA-B

## Abstract

**ОБОСНОВАНИЕ:**

ОБОСНОВАНИЕ. Сахарный диабет 2 типа (СД2) является серьезной медико-социальной проблемой. В последние годы во всем мире предпринимаются попытки новой стратификации диабета. Поэтому актуальны проведение кластерного анализа при различной длительности диабета, в разных когортах для выявления фенотипических кластеров СД2 и валидация путем воспроизведения кластеров.

**ЦЕЛЬ:**

ЦЕЛЬ. Выделить кластеры СД2 у пациентов, не получающих инсулинотерапию, на основе пяти переменных: гликированный гемоглобин (HbA1c), возраст на момент постановки диагноза, индекс массы тела (ИМТ), индекс инсулинорезистентности HOMA (HOMA-IR, ед.), индекс оценки функции β-клеток (HOMA-B) и изучить клинические особенности и частоту осложнений в каждом кластере в Новосибирской области.

**МАТЕРИАЛЫ И МЕТОДЫ:**

МАТЕРИАЛЫ И МЕТОДЫ. Кластерный анализ проведен у 2131 больного СД2 в возрасте от 44 до 70 лет, с длительностью диабета 6,42±5,66 года, проживающих в Новосибирской области, на основе 5 переменных — HbA1c, возраст на момент постановки диагноза, ИМТ, HOMA-IR, HOMA-B. Все больные проходили клиническое и лабораторное обследование. HOMA-IR и HOMA-B рассчитывали c помощью калькулятора версии 2.2.3 на сайте www.dtu.ox.ac.uk.

**РЕЗУЛЬТАТЫ:**

РЕЗУЛЬТАТЫ. По результатам кластерного анализа пациенты были отнесены к трем кластерам: кластер 1 представлен 455 пациентами с сохраненной функцией β-клеток (HOMA-B 82,97±23,28%), умеренной инсулинорезистентностью (HOMA-IR 5,57±4,72) и отличался более высоким уровнем диастолического артериального давления; кластер 2 состоял из 1658 пациентов со сниженной функцией β-клеток (HOMA-B 21,71±12,51%), наименьшими показателями инсулинорезистентности (HOMA-IR 3,50±2,48) и характеризовался большей длительностью диабета, худшей компенсацией по уровню гликемии натощак и HbA1c, более высокой расчетной скоростью клубочковой фильтрации и микроальбуминурией, мужской пол в этом кластере был ассоциирован с более высоким риском по сравнению с женщинами по развитию диабетической нейропатии (на 31%) и диабетической нефропатии (на 28%); кластер 3 представлен 18 пациентами с повышенной функцией β-клеток (HOMA-B 228,53±63,32%), выраженной инсулинорезистентностью (HOMA-IR 6,92±4,77), к особенностям данного кластера относились высокая частота мужского пола, меньшая длительность диабета, лучшая компенсация по уровню глюкозы крови натощак и HbA1c и высокая частота раннего развития диабетической ретинопатии в среднем через 4,00±3,6 года.

**ЗАКЛЮЧЕНИЕ:**

ЗАКЛЮЧЕНИЕ. На основе пяти переменных (HbA1c, возраст на момент постановки диагноза, ИМТ, HOMA-IR, HOMA-B) выделены три кластера СД2 у пациентов, не получающих инсулинотерапию: кластер 1 с сохраненной функцией β-клеток и умеренной инсулинорезистентностью, кластер 2 со сниженной функцией β-клеток и умеренной инсулинорезистентностью и кластер 3 с повышенной функцией β-клеток и выраженной инсулинорезистентностью. Кластеры имели различные клинические характеристики пациентов и частоту осложнений.

## Abstract (en)

**BACKGROUND:**

BACKGROUND: Diabetes mellitus (T2DM) is a serious medical and social problem. Now they are studying the possibility of a new stratification of diabetes. The possibility of cluster analysis for different durations of diabetes, in different cohorts to identify phenotypic clusters of T2DM and validation by reproducing clusters is relevant.

**AIM:**

AIM: Identify clusters of type 2 diabetes mellitus in patients with different disease duration based on five variables: HbA1c, age at diagnosis, BMI, HOMA-IR, HOMA-B and study the clinical features and complication rates in each cluster in the Novosibirsk region.

**MATERIALS AND METHODS:**

MATERIALS AND METHODS: Cluster analysis of K-means was performed in 2131 patients with T2DM, aged 44 to 70 years, with a duration of diabetes of 6.42±5.66 years, living in the Novosibirsk region based on 5 variables — HbA1c, age at ­diagnosis, BMI, HOMA-IR, HOMA-B. All patients a complete clinical and laboratory examination. The insulin resistance index in the HOMA (HOMA-IR, u) and the β-cell function assessment index (HOMA-B) were calculated using the calculator ­version 2.2.3 at www.dtu.ox.ac.uk.

**RESULTS:**

RESULTS: Cluster analysis revealed: Cluster 1 included 455 patients with preserved β-cell function (HOMA-B 82.97±23.28%), moderate insulin resistance (HOMA-IR 5.57±4.72) and higher diastolic BP; Cluster 2 in 1658 patients with reduced function of β-cells (HOMA-B 21.71±12.51%), the lowest indices of insulin resistance (HOMA-IR 3.50±2.48) and was characterized by a longer duration of diabetes, high fasting glycemia , HbA1c, higher eGFR and MAU, men compared with women had a 31% higher risk of developing diabetic neuropathy and 28% more diabetic nephropathy; Cluster 3 in 18 patients with high function of β-cells (HOMA-B 228.53±63.32%), severe insulin resistance (HOMA-IR 6.92±4.77), features were high incidence of men, shorter duration of diabetes, lower fasting glycemia and HbA1c, lower diastolic BP and eGFR, high incidence of early development of diabetic retinopathy after 4.00±3.6 years.

**CONCLUSION:**

CONCLUSION: Cluster analysis in patients with different durations of diabetes mellitus confirmed the possibility of using cluster analysis to identify T2DM phenotypes in the Russian population. The clusters differed in the clinical characteristics of patients, the frequency and risk of diabetic complications. These results have potential value for early stratification of therapy.

## ОБОСНОВАНИЕ

Сахарный диабет 2 типа (СД2) является серьезной медико-социальной проблемой, обусловленной развитием множественных осложнений, которые снижают качество жизни и могут привести к смерти [1]. Однако в клинической практике СД2 имеет разное клиническое течение, поэтому и выбор терапии должен быть основан на различных патогенетических механизмах, приводящих к развитию диабета. В последние годы во всем мире предпринимаются попытки новой стратификации диабета. Три подгруппы СД2 были идентифицированы с помощью топологического анализа, основанного на сетях пациент-пациент [2]. Однако эта попытка классифицировать пациентов требовала данных об их генотипе, что трудно выполнить в реальной клинической практике. В исследованиях E. Ahlqvist и соавт. на основе 6 общих клинических переменных, которые включали антитела к глютаматдекарбоксилазе (GADA), гликированный гемоглобин (HbA1c), индекс массы тела (ИМТ), возраст на момент постановки диагноза и оценки индексов инсулинорезистентности в гомеостатической модели (HOMA-IR, ед.) и функции β-клеток (HOMA-B), было выделено 5 кластеров: кластер 1 — тяжелый аутоиммунный диабет, соответствовавший СД1, кластер 2 — тяжелый диабет с дефицитом инсулина, кластер 3 — тяжелый инсулинорезистентный диабет, кластер 4 — легкий диабет, связанный с ожирением, кластер 5 — легкий диабет пожилых [3]. N. Safai и соавт. [4] использовали аналогичные клинические маркеры, чтобы разделить пациентов на 5 кластеров, и отметили разную вероятность развития осложнений диабета, таких как сердечно-сосудистые заболевания, нефропатия и нейропатия. O. Zaharia и соавт. [5] обследовали пациентов с впервые выявленным диабетом и сгруппировали их в те же 5 кластеров, последующее 5-летнее наблюдение этих больных выявило более высокую распространенность неалкогольной жировой болезни печени и нейропатии в кластере тяжелого инсулинорезистентного диабета. J. Dennis и соавт. [6] также воспроизвели 5 кластеров и установили различия в прогрессировании гипергликемии. В работе A. Kahkolska и соавт. [7] были объединены пациенты с недавно диагностированным диабетом из трех глобальных исследований (DEVOTE, LEADER, SUSTAIN-6) и проведен кластерный анализ на основе 3 переменных (возраст на момент постановки диагноза, исходный уровень HbA1c и ИМТ), индексы HOMA-IR и HOMA-В не использовали в качестве переменных, так как пациенты получали инсулинотерапию. Авторами были воспроизведены 4 кластера: тяжелый диабет с дефицитом инсулина; тяжелый инсулинорезистентный диабет; легкий диабет, связанный с ожирением, и легкий диабет пожилых. Авторами отмечено, что риск нефропатии, неблагоприятных сердечно-сосудистых исходов и смерти зависел от длительности диабета и уровня HbA1c. Самый высокий риск нефропатии, серьезных неблагоприятных сердечно-сосудистых событий и смерти от сердечно-сосудистых заболеваний был в кластере тяжелого диабета с дефицитом инсулина (с самым высоким уровнем HbA1c и низким ИМТ), а риск сердечной недостаточности — в кластере легкого диабета, связанного с ожирением. Подобные работы, в которых были воспроизведены 4 кластера СД2, исключая кластер аутоиммунного диабета, были выполнены в США и Китае [8][9]. Однако таких работ в Российской Федерации не было, кроме того, практически все исследования были выполнены на пациентах с недавно диагностированным СД. Поэтому проведение кластерного анализа при различной длительности диабета, в разных когортах диабета, включая географически разные группы населения, с целью расширения возможной области применения и разработки надежных методологий для выявления фенотипических кластеров СД2 и валидация путем воспроизведения кластеров актуальны в настоящее время.

## ЦЕЛЬ ИССЛЕДОВАНИЯ

Выделить кластеры СД2 у пациентов, не получающих инсулинотерапию, на основе 5 переменных: HbA1c, возраст на момент постановки диагноза, ИМТ, HOMA-IR, HOMA-B и изучить клинические особенности и частоту осложнений в каждом кластере.

## МАТЕРИАЛЫ И МЕТОДЫ

## Место и время проведения исследования

Место проведения. Исследование выполнено на базе передвижного лечебно-профилактического Диамодуля Государственного бюджетного учреждения здравоохранения Новосибирской области «Государственная Новосибирская областная клиническая больница» (главный врач — Юданов А.В.).

Время исследования. Исследование выполнено в период с апреля 2013 по ноябрь 2017 гг.

## Изучаемые популяции (одна или несколько)

В исследование включен 2131 пациент с СД2.

Критерии включения: СД2, возраст от 44 до 70 лет. Диагноз СД2 устанавливали по критериям Комитета экспертов Всемирной организации здравоохранения по СД (1999) и Российским клиническим рекомендациям [10].

Критерии исключения: инсулинотерапия больных СД2, наличие у больных СД1 антител к β-клеткам (ICA) и/или GADА, беременности, другие типы диабета, наличие онкологии, хронической сердечной недостаточности функциональных классов 3–4 в соответствии с классификацией Нью-Йоркской кардиологической ассоциации, хронической болезни почек (ХБП) С4–5, лечение кортикостероидами или эстрогенами, алкоголизм, наркомания, деменция или серьезные психические расстройства, острые вирусные и бактериальные инфекции.

## Способ формирования выборки из изучаемой популяции (или нескольких выборок из нескольких изучаемых популяций)

Кластерный анализ k-средних был проведен у 2131 больного СД2, проживающего в Новосибирской области, на основе 5 переменных — HbA1c, возраст на момент постановки диагноза, ИМТ, HOMA-IR, HOMA-B. Процесс очистки данных состоял из нескольких этапов: были исключены пациенты с отсутствующей информацией, а также получавшие инсулинотерапию.

## Дизайн исследования

Проведено обсервационное одновыборочное неконтролируемое динамическое исследование с ретроспективным анализом во время выездов Диамобиля в районы Новосибирской области.

## Описание медицинского вмешательства (для интервенционных исследований)

Все лечебные и диагностические вмешательства являлись частью рутинной врачебной практики.

## Методы

Больным проводили клиническое и лабораторное обследование (все были осмотрены эндокринологом, офтальмологом, кардиологом, неврологом). Осложнения устанавливали в соответствии с Российскими клиническими рекомендациями [10]. Для диагностики периферической нейропатии использовали шкалу нейропатического дисфункционального счета — НДС (Neuropathy Dysability Score). Пациентам осуществлялся забор биоматериала утром натощак с целью последующего исследования НbA1c, креатинина, холестерина общего, триглицеридов, липопротеинов низкой плотности, липопротеинов высокой плотности, аланинаминотрансферазы (АЛТ), аспартатаминотрансферазы (АСТ), с расчетом индекса фиброза печени по соотношению АСТ/АЛТ (AAR), инсулина, С-пептида и экскреции микроальбуминурии в утренней порции мочи в сертифицированной лаборатории ГБУЗ НСО «ГНОКБ». Биохимические исследования, определение инсулина, С-пептида выполняли на автоматическом анализаторе Immulite 2000. Исследование НbA1c проводили методом высокоэффективной жидкостной хроматографии на автоматическом анализаторе D-10 фирмы BIO-RAD с помощью наборов D-10 Reorder Pack, 400 Test (производства BIO-RAD LABORATORIES). HOMA-IR, ед., и HOMA-B рассчитывали c помощью калькулятора версии 2.2.3 на сайте www.dtu.ox.ac.uk. Уровень индекса HOMA-IR более 2,7 определяли как наличие инсулинорезистентности. При уровне HOMA-B более 50% функцию β-клеток считали сохраненной, более 100% — повышенной, менее 50% — сниженной.

## Статистический анализ

Кластерный анализ k-средних был проведен у 2131 больного СД2 на основе 5 переменных — HbA1c, возраст на момент постановки диагноза, ИМТ, HOMA-IR, HOMA-B. Все данные были масштабированы до среднего значения нулевой и единичной дисперсии перед кластеризацией. Количество кластеров определялось из предварительно проведенного иерархического анализа на случайно отобранных выборках (с небольшим количеством случаев). В последующем, учитывая большое количество случаев в нашей выборке и гипотезу о 3 фенотипах, в соответствии с алгоритмом Hartigan J.A., Wong M.A. применялся кластерный анализ методом k-средних (K-Means Cluster Analysis).

Для описательной статистики между группами для категорийных переменных использовали χ². Для количественных переменных при нормальном распределении данные представлены в виде среднего значения (М) и стандартного отклонения (SD). Для оценки межгрупповых различий использовался непараметрический метод Kruskal–Wallis test. Гипотеза о нормальном распределении показателей проверялась с помощью критерия Колмогорова–Смирнова при n>50, критерия Шапиро–Уилка — для n<50. Для анализа времени развития диабетических осложнений использовали анализ Каплана–Мейера. Для оценки связи между осложнениями диабета и кластерами с учетом гендерной стратификации использовали регрессию Кокса (относительный риск (ОР) (95% доверительный интервал (ДИ)). Критический уровень значимости принимали равным 0,05. Для статистической обработки использован пакет статистики SPSS 13.0.

## Этическая экспертиза

Протокол исследования одобрен комитетом по этике Новосибирского государственного медицинского университета (протокол №52, от 19.03.2013). Перед включением в исследование все пациенты подписывали информированное согласие.

## РЕЗУЛЬТАТЫ

В исследовании возраст участников варьировал от 44 до 70 лет. На основании характеристик кластеров пациенты были отнесены к 3 кластерам: кластер 1 с сохраненной функцией β-клеток и умеренной инсулинорезистентностью — 455 больных (21,4%), кластер 2 со сниженной функцией β-клеток и умеренной инсулинорезистентностью являлся самым многочисленным — 1658 больных (77,8%) и кластер 3 с повышенной функцией β-клеток и выраженной инсулинорезистентностью встречался лишь у 18 больных СД2 (0,8%). Общая характеристика популяции и клинические данные обследованных больных СД2 представлены в таблице 1.

**Table:** Таблица 1. Характеристика больных СД2 выделенных кластеров Примечание: СД — сахарный диабет, СД2 — сахарный диабет 2 типа, ИМТ — индекс массы тела, САД — систолическое артериальное давление, ДАД — диастолическое артериальное давление, рСКФ — расчетная скорость клубочковой фильтрации, МАУ — микроальбуминурия, ЛПВП — липопротеиды высокой плотности, ЛПНП — липопротеиды низкой плотности, АЛТ — аланинаминотрансфераза, АСТ — аспартатаминотрансфераза, индекс фиброза печени AAR (astartamaminotransferase to alanine aminotransferase ratio) — соотношение АСТ/АЛТ.*Статистическая значимость различий: непараметрический метод Kruskal–Wallis test.

Параметр	(кластер 1)	(кластер 2)	(кластер 3)	Р
N	455	1658	18	
Мужчины, n (%) Женщины, n (%)	138 (21,1) 317 (78,9)	350 (21,1) 1308 (78,9)	4 (22,2) 14 (77,8)	<0,001*
Возраст, лет	57,87±7,15	58,91±6,88	57,83±5,09	0,016*
Длительность СД, лет	5,85±5,39	6,60±5,74	4,0±3,53	0,008*
Возраст дебюта СД2, лет	52,14±7,92	52,33±7,97	53,83±6,17	0,645
ИМТ, кг/м²	35,26±6,47	33,82±6,48	36,61±7,54	<0,001*
Глюкоза крови натощак, ммоль/л	8,04±2,71	8,50±2,82	6,56±2,21	<0,001*
САД, мм рт. ст.	149,52±21,92	149,00±21,18	139,17±14,27	0,129
ДАД, мм рт. ст.	91,70±12,13	90,15±11,77	85,56±10,27	0,010*
рСКФ, мл/мин/1,73 м²	73,41±16,23	75,40±17,10	70,14±13,68	0,040*
Мочевая кислота, мкмоль/л	298,57±78,81	300,02±80,68	282,21±71,36	0,617
МАУ, мг/л	30,67±72,33	37,32±100,07	28,64±63,07	0,393
С-пептид, нмоль/л	1248,22±649,21	693,93±427,26	1510,78±549,32	<0,001*
Общий холестерин, ммоль/л	5,82±1,37	5,89±1,64	6,01±1,42	0,674
Триглицериды, ммоль/л	2,21±1,77	2,28±1,79	1,83±1,07	0,424
ЛПВП, ммоль/л	1,18±0,34	1,19±0,35	1,20±0,38	0,839
ЛПНП, ммоль/л	3,24±0,92	3,24±1,00	3,56±0,95	0,383
АЛТ, ЕД/л	24,96±20,83	24,73±20,41	25,67±19,97	0,962
АСТ, ЕД/л	23,20±13,51	22,98±15,26	23,03±12,87	0,963
Индекс фиброза печени AAR	3,63±1,02	3,57±0,93	3,74±1,05	0,418
HOMA-IR	5,57±4,72	3,50±2,48	6,92±4,77	<0,001*
HOMA-B	82,97±23,28	21,71±12,51	228,53±63,32	<0,001*
HbA1c	8,51±2,02	8,69±2,15	7,63±2,23	0,034*

Кластер 1 был представлен 455 пациентами с сохраненной функцией β-клеток (HOMA-B 82,97±23,28%), умеренной инсулинорезистентностью (HOMA-IR 5,57±4,72), уровнем С-пептида 1248,22±649,21 пмоль/л, HbA1c 8,51±2,02%, возрастом на момент постановки диагноза 52,14±7,92 года и ИМТ 35,26±6,47 кг/м². Данный кластер отличался более высоким уровнем диастолического артериального давления (АД), по остальным параметрам имел промежуточные значения между кластерами 2 и 3 (табл. 1).

Кластер 2, самый многочисленный, состоял из 1658 пациентов со сниженной функцией β-клеток (HOMA-B 21,71±12,51%), наименьшими показателями инсулинорезистентности (HOMA-IR 3,50±2,48) и уровня С-пептида — 693,93±427,26 пмоль/л, но более высоким HbA1c — 8,69±2,15%, возрастом на момент постановки диагноза 52,33±7,97 года и более низким ИМТ — 33,82±6,48 кг/м². Кластер 2 достоверно отличался более старшим возрастом на момент обследования — 58,91±6,88 года, большей длительностью диабета — 6,60±5,74 года, самыми высокими показателями глюкозы натощак — 8,5±2,82 ммоль/л и расчетной скорости клубочковой фильтрации (рСКФ) — 75,40±17,11 мл/мин/1,73 м², а уровень микроальбуминурии (МАУ) в данном кластере был самым высоким — 37,32±100,07 мг/л, однако данные различия были недостоверны (табл. 1). Частота макро- и микрососудистых осложнений в кластере 2 оказалась сопоставима с кластером 1 (табл. 2).

**Table:** Таблица 2. Частота осложнений и время до их развития у больных сахарным диабетом 2 типа выделенных кластеров Примечание: НДС — нейропатический дисфункциональный счет, ХБП — хроническая болезнь почек, ИБС — ишемическая болезнь сердца, ИМ — инфаркт миокарда, ЦВБ — цереброваскулярная болезнь, ХСН — хроническая сердечная недостаточность.*Статистическая значимость различий: непараметрический метод Kruskal–Wallis test.

Параметр	(кластер 1)	(кластер 2)	(кластер 3)	Р
N	455	1658	18	
Нейропатия, n (%)	202 (44,4)	798 (48,1)	11 (61,1)	0,187
Общий балл по шкале НДС, ед.	6,02±5,93	5,80±5,86	6,50±4,99	0,706
Время до развития нейропатии, лет	5,83±5,19	6,57±5,85	4,09±2,91	0,177
Нефропатия (ХБП), n (%)	155 (34,1)	526 (31,7)	5 (27,8)	0,589
Время до развития нефропатии, лет	6,08±5,52	6,67±5,85	2,40±1,14	0,077
Ретинопатия, n (%)	139 (30,5)	482 (29,1)	10 55,6)	0,044*
Время до развития ретинопатии, лет	5,43±5,10	6,45±5,50	4,00±3,06	0,037*
ИБС, n (%)	90 (19,8)	328 (19,8)	1 (5,6)	0,319
ИМ, n (%)	32 (7,0)	131 (7,9)	-	0,390
ЦВБ, n (%)	36 (7,9)	104 (6,3)	1 (5,6)	0,452
ХСН, n (%)	82 (18)	363 (21,9)	2 (11,1)	0,117

Кластер 3 представлен всего 18 пациентами с повышенной функцией β-клеток (HOMA-B 228,53±63,32%), выраженной инсулинорезистентностью (HOMA-IR 6,92±4,77), уровнем С-пептида 1510,78±549,32 пмоль/л, более низким HbA1c (7,63± 2,23%), старшим возрастом на момент постановки диагноза (53,82±6,17 года) и более высоким ИМТ (36,61±7,54 кг/м²), при этом особенностями данного кластера являлись достоверно более молодой возраст на момент обследования (57,83±5,09 года), в среднем на 2 года меньшая длительность диабета (4,0±3,53 года), более низкий уровень диастолического АД (85,56±10,27 мм рт. ст.) и рСКФ (70,14±13,68 мл/мин/1,73 м²) (табл. 1). Несмотря на малочисленность, наименьшую длительность диабета, лучшие показатели компенсации углеводного обмена и более низкий уровень диастолического АД, кластер 3 (с повышенной функцией β-клеток и выраженной инсулинорезистентностью) отличался наибольшей частотой диабетической ретинопатии (55,6% против 30,5% (при кластере 1) и 29,1% (при кластере 2)) и более ранним ее развитием (в среднем через 4,00±3,6 года по сравнению с кластером 1 — 5,43±5,10 года и кластером 2 — 6,45±5,50 года) (табл. 2).

При анализе времени развития диабетической ретинопатии методом Каплана–Майера также было подтверждено, что при кластере 3 диабетическая ретинопатия развивается быстрее по сравнению с кластерами 1 и 2 (χ²=6,833; р=0,033) (рис. 1).

**Figure:**
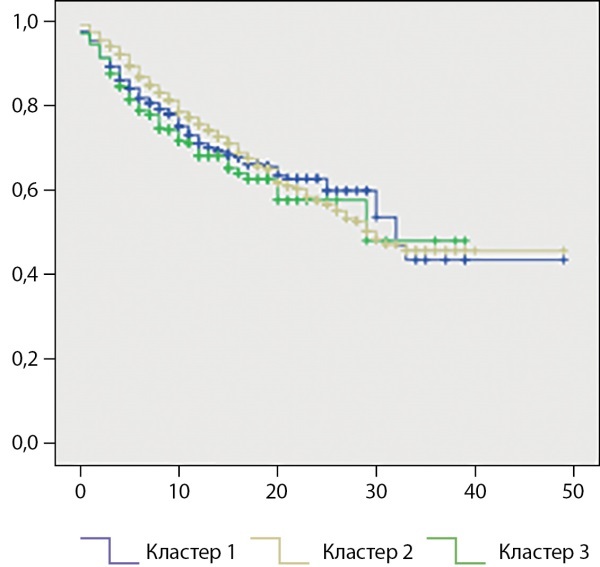
Рисунок 1. Время до развития диабетической ретинопатии при различных кластерах сахарного диабета 2 типа (кривая Каплана–Майера).ось Х — время в годах (от момента диагностики СД до момента верификации осложнения), ось Y — доля пациентов без осложнений (1,0 — осложнения нет).

Была изучена связь между осложнениями диабета и кластерами с учетом гендерной стратификации. Было установлено, что только при кластере 2 со сниженной функцией β-клеток и умеренной инсулинорезистентностью риск развития диабетической нейропатии (ОР (95% ДИ) 0,690 (0,544; 0,875), р=0,002) и диабетической нефропатии (ОР (95% ДИ) 0,723 (0,565; 0,926), р=0,010) был ниже у женщин по сравнению с мужчинами (табл. 3).

**Table:** Таблица 3. Риск развития осложнений в различных кластерах сахарного диабета 2 типа с учетом пола Примечание: Регрессионный анализ Кокса. ОР — относительный риск, ДИ — доверительный интервал, ИБС — ишемическая болезнь сердца, ИМ — инфаркт миокарда, ЦВБ — цереброваскулярная болезнь, ХСН — хроническая сердечная недостаточность.

Осложнение	Кластер 1	ОР (95% ДИ)	Р
Мужчины (n=138)	Женщины (n=317)
Нейропатия	58 (42,0)	144 (45,4)	1,148 (0,767; 1,719)	0,503
Нефропатия	49 (35,5)	106 (33,4)	0,912 (0,600; 1,388)	0,669
Ретинопатия	38 (27,5)	101 (31,9)	1,231 (0,791; 1,914)	0,357
ИБС	31 (22,5)	59 (18,6)	0,789 (0,484; 1,288)	0,343
ИМ	9 (6,5)	23 (7,3)	1,121 (0,505; 2,490)	0,778
ЦВБ	12 (8,7)	24 (7,6)	0,860 (0,417; 1,774)	0,683
ХСН	23 (16,7)	59 (18,6)	0,875 (0,515; 1,485)	0,620
Осложнение	Кластер 2	ОР (95% ДИ)	Р
Мужчины (n=350)	Женщины (n=1308)
Нейропатия	194 (55,4)	604 (46,2)	0,690 (0,544; 0,875)	0,002*
Нефропатия	131 (37,4)	395 (30,2)	0,723 (0,565; 0,926)	0,010*
Ретинопатия	109 (31,1)	373 (28,5)	0,882 (0,683; 1,139)	0,337
ИБС	80 (22,9)	248 (19,0)	0,790 (0,594; 1,050)	0,104
ИМ	33 (9,4)	98 (7,5)	0,778 (0,515; 1,176)	0,233
ЦВБ	24 (6,9)	80 (6,1)	0,885 (0,552; 1,419)	0,612
ХСН	82 (23,4)	281 (21,5)	1,118 (0,845; 1,480)	0,434
Осложнение	Кластер 3	ОР (95% ДИ)	Р
Мужчины (n=4)	Женщины (n=14)
Нейропатия	3 (75,0)	8 (57,1)	0,444 (0,037; 5,406)	0,518
Нефропатия	2 (50,0)	3 (21,4)	0,273 (0,026; 2,829)	0,261
Ретинопатия	2 (50,0)	8 (57,1)	1,333 (0,144; 12,369)	0,800
ИБС	1 (25,0)	0	-	-
ИМ	0	0	-	-
ЦВБ	1 (25,0)	0	-	-
ХСН	0 (0)	2 (14,3)	-	0,423

Сахароснижающая терапия была представлена следующими вариантами: монотерапия метформином (МФ) — у 624 (29,3%) пациентов, монотерапия препаратами сульфонилмочевины (СМ) — у 153 человек (7,2%) и комбинированная терапия МФ+СМ — 1084 (50,1%) больных СД2. При сравнении ответа на сахароснижающую терапию, которую оценивали по достижению целевого уровня HbA1с (менее 7,0%), при различных кластерах СД2 было отмечено, что при кластере 3 (с повышенной функцией β-клеток и выраженной инсулинорезистентностью) достоверно чаще регистрировался хороший ответ на монотерапию СМ (27,8% по сравнению с кластером 1 (с сохраненной функцией β-клеток и умеренной инсулинорезистентностью) — 7,3% и кластером 2 (со сниженной функцией β-клеток и умеренной инсулинорезистентностью) — 5,3% (р<0,0001)), а комбинированная терапия МФ и СМ достоверно чаще позволяла достигать целевого HbA1с при кластерах 1 и 2 по сравнению с кластером 3. Достоверных различий частоты использования монотерапии МФ и ее эффективности в различных кластерах СД2 не отмечено (р=0,217) (табл. 4).

**Table:** Таблица 4. Сравнение применения препаратов в различных кластерах сахарного диабета *Статистическая значимость различий: непараметрический метод Kruskal–Wallis test. МФ — метформин, СМ — сульфонилмочевина.

Препарат	Кластер 1	Кластер 2	Кластер 3	Р
n=455	n=1658	n=18
Монотерапия МФ хороший ответ плохой ответ	141 (31,0) 82 (18,0) 59 (13,0)	477 (28,8) 288 (17,4) 189 (11,4)	6 (33,3) 6 (33,3) 0	0,217
Монотерапия СМ хороший ответ плохой ответ	109 (23,9) 33 (7,3) 76 (16,7)	308 (18,6) 88 (5,3) 220 (13,3)	6 (33,3) 5 (27,8) 1 (5,6)	<0,01
Комбинированная терапия МФ+СМ хороший ответ плохой ответ	205 (45,1) 58 (12,7) 147 (29,0)	873 (52,6) 215 (13,0) 658 (37,6)	6 (33,3) 2 (11,1) 4 (22,2)	0,006

## ОБСУЖДЕНИЕ

## Репрезентативность выборок

Клиническая характеристика обследованных нами больных согласуется с данными других эпидемиологических исследований [11] и отражает в целом популяцию больных СД2, что в определенной степени позволяет экстраполировать полученные нами результаты на целевую популяцию.

## Сопоставление с другими публикациями

В проведенном исследовании, включавшем больных СД2, проживающих в Новосибирской области, подход, основанный на кластерном анализе, был воспроизводимым, а распределение на кластеры отличалось от шведской и китайской когорты [3][9], что можно объяснить исключением из нашего анализа пациентов в возрасте старше 70 лет и пациентов на инсулинотерапии. Для возможности применения данного кластерного подхода в клинических условиях исследование включало пациентов не только с недавно диагностированным, но и с длительно существовавшим диабетом, что отличает данную работу от предыдущих исследований, которые охватывали только лиц с недавно диагностированным диабетом [3–6]. Лишь в исследование китайских авторов, так же как и в нашей работе, были включены пациенты с различной длительностью диабета [9]. Особо следует отметить, что клинические характеристики кластеров в этом исследовании были аналогичны предыдущим исследованиям, с большей долей пациентов в кластере тяжелого инсулинодефицитного диабета (77,8%) и меньшей долей тяжелого инсулинорезистентного диабета (0,8%). Это несоответствие может быть результатом включения большой когорты больных с длительным диабетом, а также этнических особенностей, что объясняет более высокую частоту тяжелого инсулинорезистентного диабета в китайской популяции (21%) [9], кроме того, в отличие от китайских исследователей, которые включили в анализ 40% больных СД2 на инсулинотерапии [9], из данной работы были исключены пациенты, получавшие инсулин.

Самая низкая рСКФ у пациентов с выраженной инсулинорезистентностью согласуется с результатами других исследований, а возможным объяснением является резистентность к инсулину, которая может привести к задержке воды и натрия, клубочковой гипертензии, гиперфильтрации и гиперурикемии, что может ускорить прогрессирование ХБП [9][12]. Нами также отмечено, что мужской пол ассоциирован с более высоким риском нефропатии — на 28% и нейропатии — на 31% в кластере со сниженной функцией β-клеток и умеренной инсулинорезистентностью, что соответствует диабету с тяжелым инсулинодефицитом у других авторов. Повышенный риск ХБП у мужчин и более медленное снижение СКФ и лучшая выживаемость пациентов женского пола с ХБП были продемонстрированы в 10-летнем исследовании [13]. В 2017 г. K. Matsushita с соавт., обобщив многочисленные публикации, сообщили, что риск смертности от ХБП у мужчин выше, чем у женщин [14].

Диабетическая ретинопатия встречалась у 30% пациентов с СД [15], в работе китайских авторов чаще регистрировалась в кластере с дефицитом инсулина (32%) и диабетом с умеренной инсулинорезистентностью и ожирением (32%) [9]. Нами выявлена аналогичная частота ретинопатии в кластере с дефицитом инсулина — 29,1% и с умеренной инсулинорезистентностью и ожирением — 30,5%, однако, в отличие от исследования китайских ученых, наибольшая частота диабетической ретинопатии (55,6%) и более раннее ее развитие (в среднем через 4,00±3,6 года) нами были отмечены в группе с выраженной инсулинорезистентностью. Многочисленные исследования доказали, что гипергликемия и артериальная гипертензия являются факторами риска ретинопатии за счет усиленного гликозилирования, повышения экспрессии провоспалительных молекул в сетчатке [16]. Согласно литературным данным, ретинопатия часто может предшествовать диабетической нефропатии у пациентов с СД2 [15].

## Клиническая значимость результатов

Проведенное исследование показало, что кластерный анализ у пациентов как при недавно выявленном диабете, так и при различной длительности диабета может дать стабильный результат, и подтвердило возможность применения кластерного анализа для выделения фенотипов СД2 в российской популяции. Каждый кластер имел различные клинические характеристики пациентов и частоту диабетических осложнений. Эти результаты имеют потенциальную ценность для последующих исследований и ранней стратификации терапии.

## Ограничения исследования

Наше исследование имеет несколько важных недостатков: умеренный размер выборки, включение пациентов с различной длительностью СД2 и исключение пациентов на инсулинотерапии, что может влиять на размер кластеров, увеличивая количество пациентов в кластере со сниженной функцией β-клеток и уменьшая количество больных в кластере с выраженной инсулинорезистентностью и повышенной функцией β-клеток.

## Направления дальнейших исследований

С целью изучения риска и времени развития осложнений и исходов в зависимости от клинического фенотипа СД2 продолжается наблюдение за включенными в исследование пациентами.

## ЗАКЛЮЧЕНИЕ

На основе 5 переменных (HbA1c, возраст на момент постановки диагноза, ИМТ, HOMA-IR, HOMA-B) выделено 3 кластера СД2 у пациентов, не получающих инсулинотерапию: кластер 1 с сохраненной функцией β-клеток и умеренной инсулинорезистентностью отмечен у 455 больных (21,4%), кластер 2 со сниженной функцией β-клеток и умеренной инсулинорезистентностью — у 1658 больных (77,8%), кластер 3 с повышенной функцией β-клеток и выраженной инсулинорезистентностью — у 18 больных СД2 (0,8%). Кластер 1 с сохраненной функцией β-клеток и умеренной инсулинорезистентностью характеризовался более высоким уровнем диастолического АД; кластер 2 со сниженной функцией β-клеток и наименьшими показателями инсулинорезистентности отличался более старшим возрастом на момент обследования, большей длительностью диабета, самыми высокими показателями глюкозы натощак и рСКФ; кластер 3 с повышенной функцией β-клеток и выраженной инсулинорезистентностью был самым малочисленным, имел наименьшую длительность диабета, лучшие показатели компенсации углеводного обмена, однако отличался более низкой рСКФ и более высокой частотой диабетической ретинопатии (55,6% против 30,5% (при кластере 1) и 29,1% (при кластере 2)).

## ДОПОЛНИТЕЛЬНАЯ ИНФОРМАЦИЯ

Источники финансирования. Исследование выполнено при поддержке гранта РФФИ 13-04-00520.

Конфликт интересов. Авторы декларируют отсутствие явных и потенциальных конфликтов интересов, связанных с содержанием настоящей статьи.

Участие авторов. Бондарь И.А.: 1 — существенный вклад в концепцию исследования; 2 — в интерпретацию результатов исследования; 3 — внесение в рукопись существенной правки с целью повышения научной ценности статьи; Шабельникова О.Ю.: 1 — существенный вклад в концепцию исследования; 2 — в получение, анализ данных и интерпретацию результатов исследования; 3 — написание статьи. Все авторы одобрили финальную версию статьи перед публикацией, выразили согласие нести ответственность за все аспекты работы, подразумевающую надлежащее изучение и решение вопросов, связанных с точностью или добросовестностью любой части работы.

Благодарности. Авторы выражают благодарность профессиональному математику Лилии Валерьевне Щербаковой, сотруднику Научно-исследовательского института терапии и профилактической медицины — филиала Федерального государственного бюджетного научного учреждения «Федеральный исследовательский центр Институт цитологии и генетики СО РАН» за помощь в проведении кластерного анализа.
